# Assessment of Food Supplement Consumption in Polish Population of Adults

**DOI:** 10.3389/fnut.2021.733951

**Published:** 2021-10-27

**Authors:** Katarzyna Stoś, Agnieszka Woźniak, Ewa Rychlik, Izabela Ziółkowska, Aneta Głowala, Maciej Ołtarzewski

**Affiliations:** Department of Nutrition and Nutritional Value of Food, National Institute of Public Health NIH - National Research Institute, Warsaw, Poland

**Keywords:** food supplements, vitamins, minerals, vitamin intake, mineral intake

## Abstract

**Introduction:** In recent years, there has been a great interest in food supplements. However the use of food supplements can be associated with the risk of excessive intake of vitamins or minerals which may have adverse health effects.

**Objective:** Assessment of food supplement consumption in the adult population in Poland.

**Materials and Methods:** The study was conducted on 1,831 adults (913 men, 918 women) from which 178 (59 men, 119 women) food supplement users were selected. The consumption of food supplements were assessed by the 24-h recall repeated two times and the food propensity questionnaire (FPQ).

**Results:** 10% of the subjects consumed food supplements during the 12 months prior to the study (6% of men, 13% of women) and among users 68% (79% of men and 88% of women) in the day before the survey. Most respondents (44%) used vitamin supplements during the year. More men than women (27 vs. 11%, *p* = 0.0059) used mineral supplements while more women than men used vitamin and mineral supplements (31 vs. 8%, *p* = 0.0008). The most frequently supplemented vitamins were: B_6_ (58%), C (53%), and D (47%) and minerals were: magnesium (43%), zinc (34%), and iron (29%). More women than men supplemented vitamin B6 (71 vs. 40%, *p* = 0.0012), vitamin D (54 vs. 36%, *p* = 0.0061) and magnesium (49 vs. 34%, *p* = 0.0075). Intake of riboflavin, pantothenic acid and manganese were higher in the group of men (respectively: 3.3 mg ± 6.0 vs. 1.4 mg ± 0.3, *p* = 0.0329; 9.4 mg ± 5.6 vs. 6.1 mg ± 2.0, *p* = 0.0357; 2.2 mg ± 0.9 vs. 1.3 mg ± 0.6, *p* = 0.0080) but intake of vitamin D was higher in the group of women (15.7 μg ± 20.4 vs. 33.1 μg ± 26.4, *p* = 0.0085). In many cases, the intake of vitamins and minerals from food supplements covered the Dietary Reference Values for these nutrients in 100%. In some persons the intake of biotin, vitamin B_12_, C, B_6_, riboflavin, niacin was higher than the reference values several dozen times. The intake of vitamins and minerals exceed UL in a few cases relating to vitamin B_6_ and magnesium.

**Conclusions:** A minority of adults in Poland used food supplements. However, those products were a significant source of vitamins and minerals. Intake of vitamins and minerals from food supplements should be monitored.

## Introduction

Diet is one of the key factors determining human health and it should cover the organism's need for nutrients. Well balanced diet, rich in fruits and vegetables, that are source of vitamins and other nutrients may reduce the risk of chronic diseases, such as cardiovascular diseases, cancers, diabetes and osteoporosis (1, 2). In turn, micronutrient deficiency can have wide-range negative health impacts (3). The WHO states, that the best way of preventing micronutrient malnutrition is to ensure consumption of a balanced diet that is adequate in every nutrient (4). To help people eat better, healthy eating guidelines have been developed in many countries around the world (5). They provide evidence-based statements on food choices to meet nutritional requirements and reduce the risk of prevailing chronic disease (6). However, recommendations for taking food supplements (foodstuff which are concentrated sources of nutrients i.e., minerals and vitamins or other substances with a nutritional or physiological effect) have also been developed for certain population groups, where scientific evidence indicates a benefit of such supplementation. For example, it is commonly recommended to supplement diet with vitamin D in all population groups of Central Europe countries, including Poland (7, 8). Other example are pregnant women who have increased requirements for certain nutrients, like folic acid, supplementation of which is recommended in order to reduce the risk of neural tube defects in infants (9). An elderly people may also need supplementation, as absorption of some nutrients becomes less efficient with aging, but the need for the supplementation should be confirmed by a doctor (10, 11). For vegans, vitamin B_12_ must be obtained from regular use of vitamin B_12_-fortified foods, otherwise a daily vitamin B_12_ supplement is needed (12).

However, the constantly expanding market of supplements and their non-prescription status make them generally available for all population groups (13). Young people who consume an unbalance diet, instead of improving eating habits, use food supplements with the belief that it will reduce the risk of serious health complications (14, 15). Adult food supplement users perceive supplements as an easy and safe way to stay healthy (16, 17). Although the issues related to the composition and labeling of food supplements have been legally regulated at the EU level (18), there are still elements for which no legislative solutions have been developed. In Poland, the provisions of Directive 2002/46/EC are implemented by the Food and Nutrition Safety Act (19) and the Regulation of the Minister of Health on the composition and labeling of food supplements (20). The law defines, inter alia, what vitamins and minerals and what their chemical forms may be used in the food supplements. However, there are still no maximum levels of vitamins and minerals in supplements set at the EU level. The law only says what criteria should be taken into account when setting such levels. These are: upper safe levels of vitamins and minerals established by scientific risk assessment based on generally accepted scientific data, taking into account, as appropriate, the varying degrees of sensitivity of different consumer groups, the intake of vitamins and minerals from other dietary sources and the reference intakes of vitamins and minerals for the population (18, 20). The lack of regulations in the above scope makes it possible to use high doses of vitamins and minerals in food supplements, and unjustified consumption of vitamin and mineral products may lead to exceeding the safe levels of these nutrients (21, 22). In that context the food supplement consumption in the adult population in Poland was assessed. Firstly it was estimated what percentage of Polish adults consumed food supplements. Then, the detailed examination only among people who reported the consumption of these products was made. In order to check if there was a risk of excessive intake of vitamins and minerals only from food supplements the composition of food supplements used by the subjects as well as the amounts of vitamins and minerals they consumed within these products were analyzed. These amounts have been compared to the Dietary Reference Values and Tolerable Upper Intake Levels (UL) in order to assess whether food supplements were a significant and safe source of these nutrients. The result on intake of vitamins and minerals from all dietary sources including base diet will be analyzed and published separately.

## Materials and Methods

### Study Population

The data was collected as part of the national dietary cross-sectional survey in Poland on the representative sample of the adolescent and adult population which was conducted from July 2019 to February 2020. The survey included assessment of nutrition, nutritional status and physical activity and was carred out according to the EFSA guidance on the EU Menu methodology (23, 24).

The study was conducted after obtaining the approval of the Bioethics Committee at the Institute of Food and Nutrition in Warsaw. Participation in the study was voluntary, each respondent gave the written consent to participate in it.

The sample selection for the study was done by the stratified sampling method using the PESEL (In Polish: Powszechny Elektroniczny System Ewidencji Ludności) system taking into account such demographic details as age, gender and place of residence. There were two parts of the sample selection procedure: the stratification of the Polish population and the random selection of individuals. Data was collected in all 16 voivodeships of Poland. The number of subjects living in cities and villages was corresponding to the structure in the particular voivodeship. The following exclusion criteria for the participation in the study were adopted:

- subjects that were hospitalized and/or were following an enteral and parenteral nutrition because of their health conditions,
- subjects, whose mental condition made impossible to obtain reliable information (neurodegenerative diseases, drunkenness, state after taking drugs and other stimulating substances).

The number of subjects selected using a stratified sampling method was 10 times higher than the one assumed, in order to prevent unforeseen circumstances or refusal and ensure the planned number of people to be finally studied. If a selected person refused to participate in the survey, withdrew from the study or met the criteria for exclusion from the study, the next person in the group was chosen. Only subjects who participated in all parts of the study were included. The interviewers were obliged to make repeated attempts to contact the randomly selected respondent until the interview was successfully completed or the definitive inability to complete it was confirmed (hard refusal, prolonged absence of the respondent during the survey). Attempts were made to contact 4,249 persons. The study involved 57% of persons who were invited (response rate). Subjects that fall under the exclusion criteria was not taken into account when calculating the above mentioned response rate.

The complete set of data was collected from 2,432 adolescents and adults (1,214 males and 1,218 females). There were 1,831 adults (913 men and 918 women) in that group. Pregnant or breastfeeding women were not subjects of the study. From among adults, persons who declared in the survey that they use food supplements were selected (213 persons). We excluded 35 participants because the products they had indicated as food supplements were drugs, not food supplements.

Therefore, for the assessment of food supplement consumption 178 adults (59 men, 119 women) were included.

### Data Collection

The survey methodology followed the EFSA EU Menu guidance recommendations (23, 24). Socio-demographic data like age, gender, place of residence, education level, labor activity, economic status (respondent's self-assessment) and health status (respondent's self-assessment) were collected using the questionnaire. For this purpose, the interviewers used the CAPI technique (Computer Assisted Personal Interview).

The consumption of food supplements was assessed by the food propensity questionnaire (FPQ) and 24-h recall repeated two times to obtain both data on the consumption of food supplements during the 12 months prior to the study and data on the current consumption of these products (in the day before the survey).

The FPQ included questions about the food supplement consumption during the 12 months prior to the study. Respondents were asked about trade name, brand, manufacturer and composition of consumed supplements. They also defined how often particular food supplements were consumed during the 12 months prior to the study (every day, a few times a week, few times a month, periodically or occasionally).

Questions on food supplement consumption were also included in the 24 h recall which was used to collect food consumption data. The dietary recall included information about the time and place of consumption of the meal, kind of the meal and portion size. Interviewers also asked if the respondent consumed any supplements in the last day, if yes, respondent was asked about its name, brand, producer, composition, as well as a single dose. The data was entered into the Internet platform software by interviewers. Only data on the consumption of food supplements were used in this study. The consumption of other food products was not analyzed.

In the case of multi-component food supplements or consumption of several products, it was very helpful to take by interviewers pictures of the packaging for consumed food supplements (if the respondent had it) and completed data after interview.

The questionnaire on socio-demografic details and FPQ were completed during the first interview at respondent's home. The first 24-h recall was also conducted during this visit. The second interview was conducted a minimum of 7 days but no longer than 14 days after the first interview, in such a way as to cover equal proportion of all days in the week. Data on vitamins and minerals intake from food supplements were assessed by calculating the mean across two days from 24-h dietary recall.

Due to the composition, food supplements consumed by subjects were divided into 5 groups: “vitamins only”, “minerals only”, “vitamins and minerals”, “vitamins and/or minerals and other ingredients” and “other” (supplements containing ingredients other than vitamins and minerals). The other ingredients were, such as: fish oils, omega 3 fatty acids, plant ingredients (citrus bioflavonoids, lutein, lycopene, linseed oil, *Oenothera* oil, extracts of various plants, among others: green tea, ginger, dandelion, guarana, *Ginkgo biloba, Panax ginseng, Rosa canina, Equisetum arvense, Morus alba, Uva-ursi, Urtica dioica, Yerba mate, Plantago ovata*), amino acids (L-alanine, L-arginine, L-aspartic acid, L-cysteine, L-glutamic acid, L-glycine, L-histidine, L-isoleucine, L-leucine, L-methionine), collagen, hyaluronic acid.

The intake of vitamins and minerals by each person was compared with Dietary Reference Values for the Polish population (25) to Estimated Average Requirement (EAR) or Adequate Intake (AI), regarding age and gender and also to Tolerable Upper Intake Levels (UL) established for these nutrients by European Food Safety Authority (EFSA) (21, 26–28).

### Statistical Analysis

The results were statistically tested using a computer software PQStat 1.8.2. In order to verify whether the distribution was normal, the *Shapiro-Wilk* test was used. The data distributions weren't normal so the significance of differences was assessed using the *Mann-Whitney U* test for non-parametric data and the chi-square test for qualitative data. For all analyses, the significance level α = 0.05 was assumed. Relationships between the gender and other parameters were examined using the Spearman's correlation (non-parametric data).

## Results

### Characteristic of Subjects

Ten percent of the 1,831 subjects consumed food supplements (6% of men and 13% of women) during the 12 months prior to the study. The average age of subjects consuming food supplements was 54.8 ± 19.5 (18–96) years and it was similar in men and women (55.9 ± 20.3 (18–89) vs. 54.2 ± 19.1 (18–96), *p* = 0.3857). The greatest number of men consuming such products was recorded in the age group of 65 years and over (42%) while the greatest number of women consuming food supplements was observed in the age group of 18–30 years (35%). However, that difference was not statistically significant. Most of the respondents had an upper secondary education (42%) and they were mainly retirees and pensioners (46%). Compared to women, men were more likely to work physically and less mentally (*p* = 0.0076). The economic status of the greatest number of respondents was “neither good nor bad” (52%) or “good” (35%). Also, many subjects described their health status as “neither good nor bad” (39%) or “good” (39%). There were no statistically significant differences between men and women both in case of economic status and health status (Table 1).

**Table 1:** Characteristic of subjects.

**Characteristic**	**Total**		**Men**		**Women**		**Men vs. women** *p*-value^*^
	***n* = 178**	**%**	***n* = 59**	**%**	***n* = 119**	**%**	
**Age (years):**							
18–30	53	30	11	19	42	35	0.6964
31–50	42	24	12	20	30	25	
51–64	38	21	11	19	27	23	
>=65	45	25	25	42	20	17	
**Education level:**							
Primary education/lower secondary education	14	8	5	8	9	8	0.2565
Vocational education	51	29	22	37	29	24	
Upper secondary education	74	42	24	41	50	42	
Post-secondary education	7	4	1	2	6	5	
High education	32	18	7	12	25	21	
**Labor activity:**							
Student	3	2	2	3	1	1	0.0076
Phisical work	26	15	16	27	10	8	
Mental work	54	30	14	24	40	34	
Fulfilling domestic tasks	8	4	0	0	8	7	
Uneployed	2	1	1	2	1	1	
Retire/pensioner	82	46	26	44	56	47	
Others	2	1	0	0	2	2	
**Economic status:**							
Bad	10	6	4	7	6	5	0.7856
Neither good nor bad	93	52	29	49	64	54	
Good	63	35	22	37	41	34	
Very good	12	7	4	7	8	7	
**Health status:**							
Bad	5	3	3	5	2	2	0.1457
Neither good nor bad	70	39	21	36	49	41	
Good	70	39	25	42	45	38	
Very good	33	19	10	17	23	19	

* *chi-square test*.

### Food Supplement Consumption During the 12 Months Prior to the Study

The greatest number of subjects (76% in total, 80% of men and 75% of women) used only one food supplement but a significant percentage of subjects (18% in total, 14% of men and 20% of women) declared consumption of two such products during the 12 months prior to the study. From 1 to 2% of subjects in total reported consuming more food supplements (3–9 products). Most men and women consumed food supplements every day. Much fewer subjects reported taking supplements several times a week. There were no statistically significant differences between men and women in the frequency of consumption of food supplements during 12 months prior to the study (Table 2).

**Table 2:** Frequency of food supplement consumption during the 12 months prior to the study.

**Frequency**	**Total**		**Men**		**Women**		**Men vs. women**
	***n* = 178**	**%**	***n* = 59**	**%**	***n* = 119**	**%**	*p*-value^*^
Every day	126	71	45	77	81	68	0.1961
A few times a week	26	16	7	12	17	14	0.4657
Several times a month	9	5	2	3	7	6	0.8278
Periodically	10	6	2	3	8	7	0.3633
Occasionally	9	5	3	5	6	5	0.6355

* *chi-square test*.

**Figure 1:**
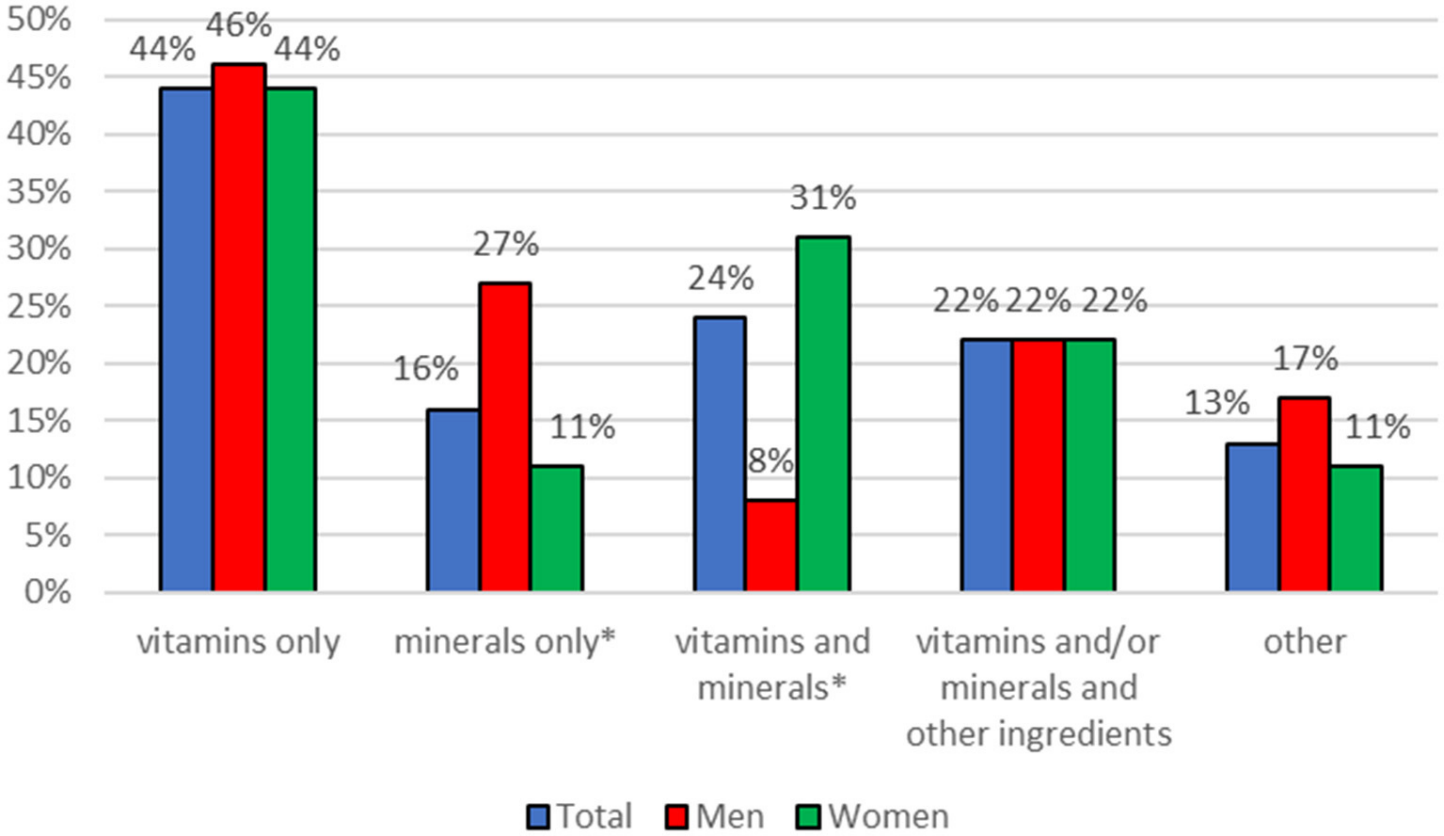
Type of food supplements consumed during the 12 months prior to the study. ^*^statistically significant difference, chi-square test, *p* < 0.05.

Most respondents used vitamin supplements during the year. A significant relationship was observed between gender and the consumption of mineral food supplements (rs = −0.2064, *p* = 0.0057) and vitamin and mineral food supplements (rs = 0.2507, *p* = 0.0007). More men than women (27 vs. 11%, *p* = 0.0059) used mineral supplements while more women than men used vitamin and mineral supplements (31 vs. 8%, *p* = 0.0008) (Figure 1).

### Food Supplement Consumption in the Day Before the Survey

**Figure 2:**
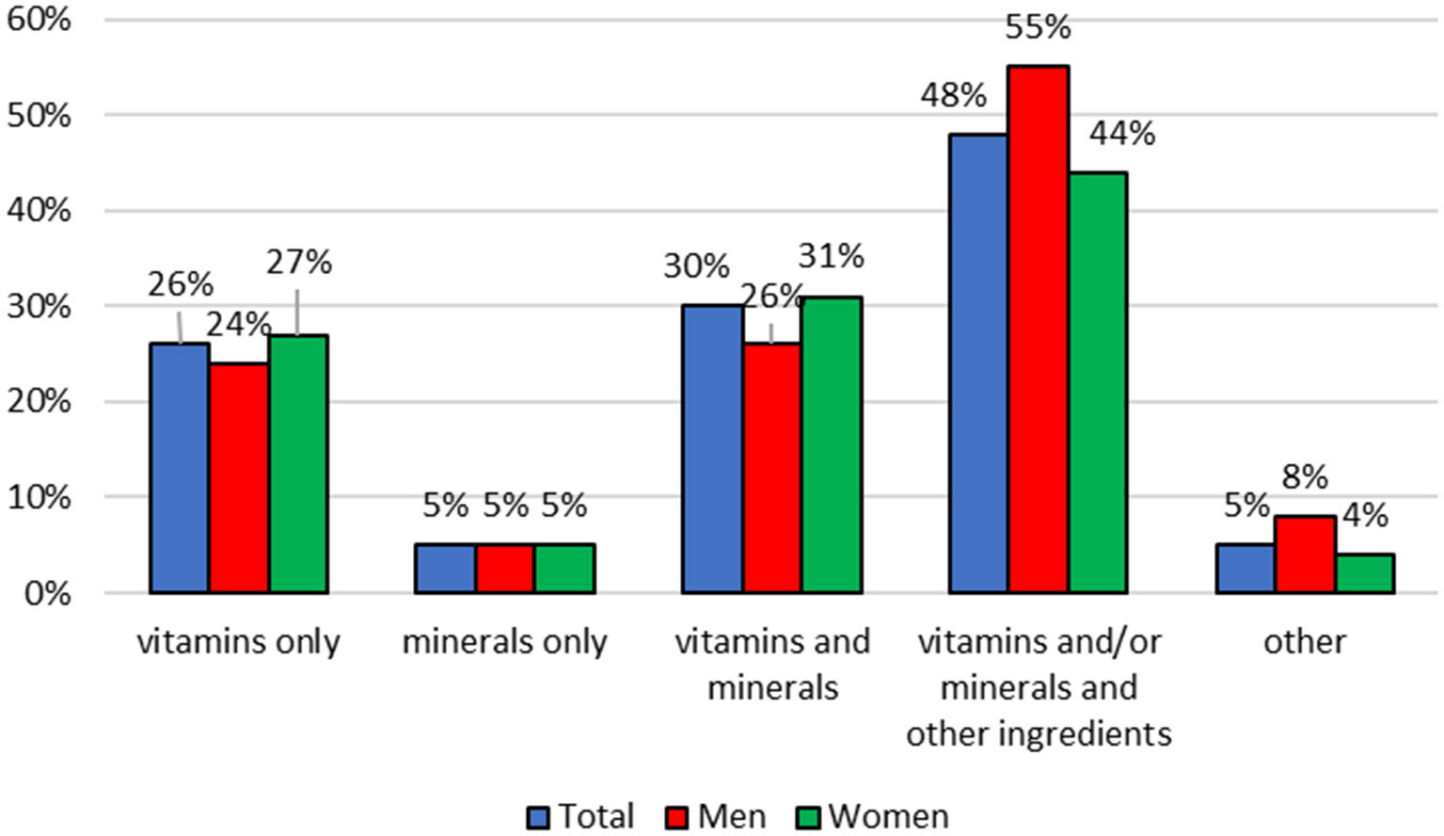
Type of food supplements consumed in the day before the survey.

Among subjects using food supplements, 68% (79% of men and 88% of women) declared consumption of these products in the day before the survey. The most of the respondents used one product (86% in total, 79% of men, 88% of women) but some consumed 2 (respectively: 10, 16, and 7%) or three (respectively: 4, 5, and 4%) products a day. The food supplements containing vitamins and/or minerals and other ingredients were consumed the most frequently in the day before the survey. T There were no statistically significant differences between percentage of men and women consuming particular types of food supplements (*p* ≥ 0.05) (Figure 2).

### Assessment of Vitamin and Mineral Intake From Food Supplements

**Figure 3:**
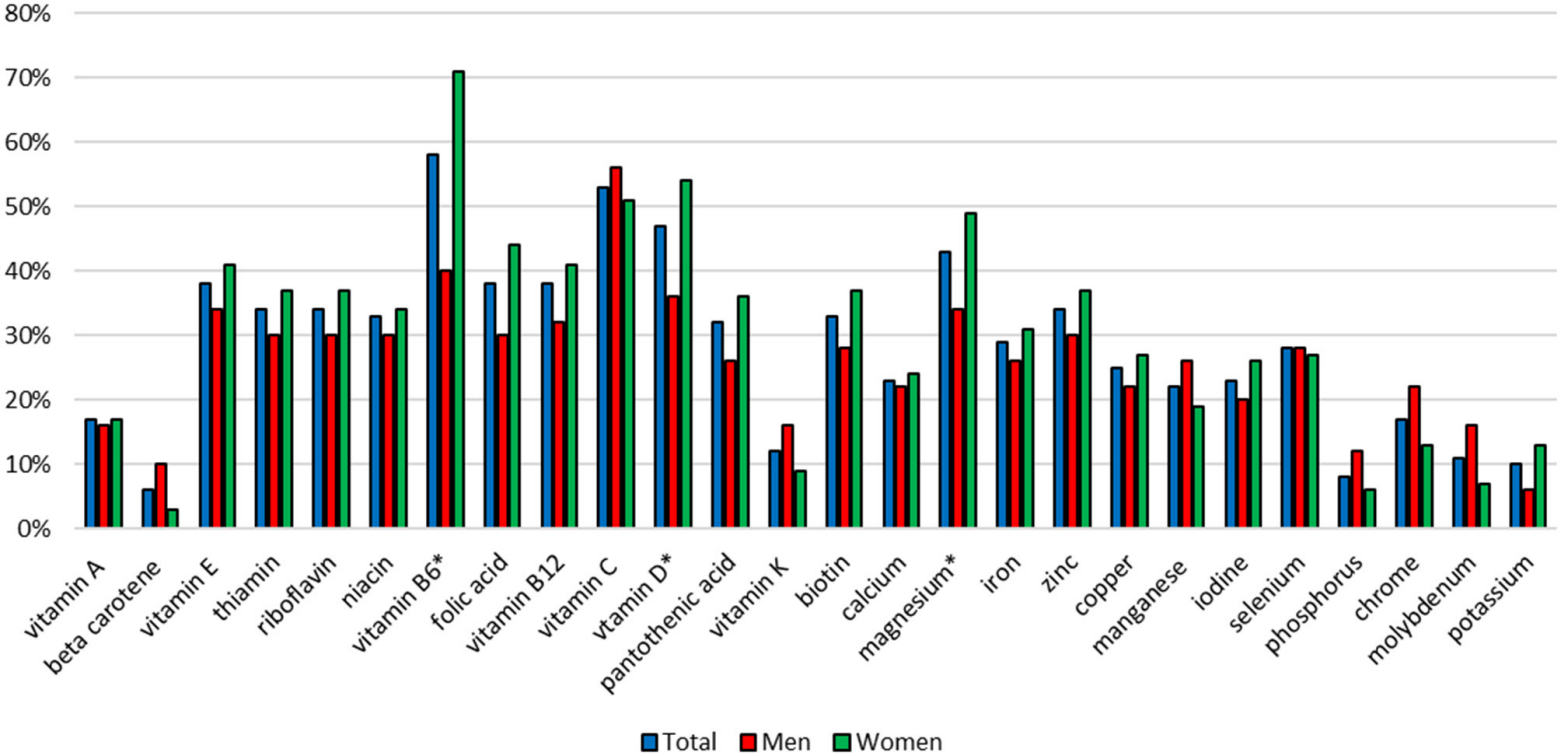
Percentage of subjects supplementing particular vitamins and minerals. *statistically significant difference, chi-square test, *p* < 0.05.

In case of vitamins, the most subjects supplemented vitamin B_6_ (58%), vitamin C (53%) and vitamin D (47%). The most frequently supplemented minerals were magnesium (43%), zinc (34%) and iron (29%). More women than men supplemented vitamin B_6_ (71 vs. 40%, *p* = 0.0012), vitamin D (54 vs. 36%, *p* = 0.0061) and magnesium (49 vs. 34%, *p* = 0.0075) (Figure 3).

**Figure 4:**
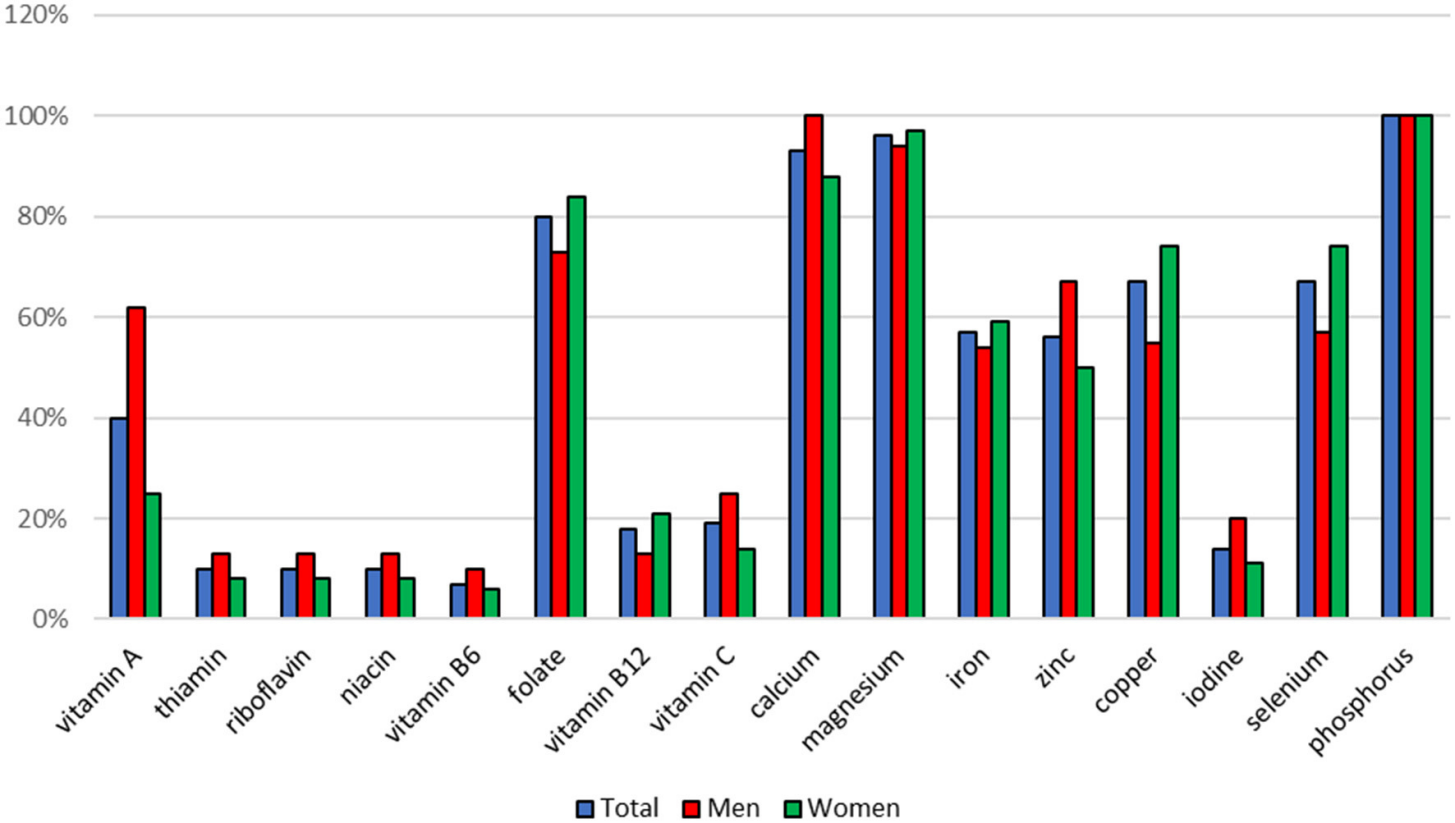
Percentage of people whose intake of vitamins and minerals from food supplements was lower than the EAR value.

**Figure 5:**
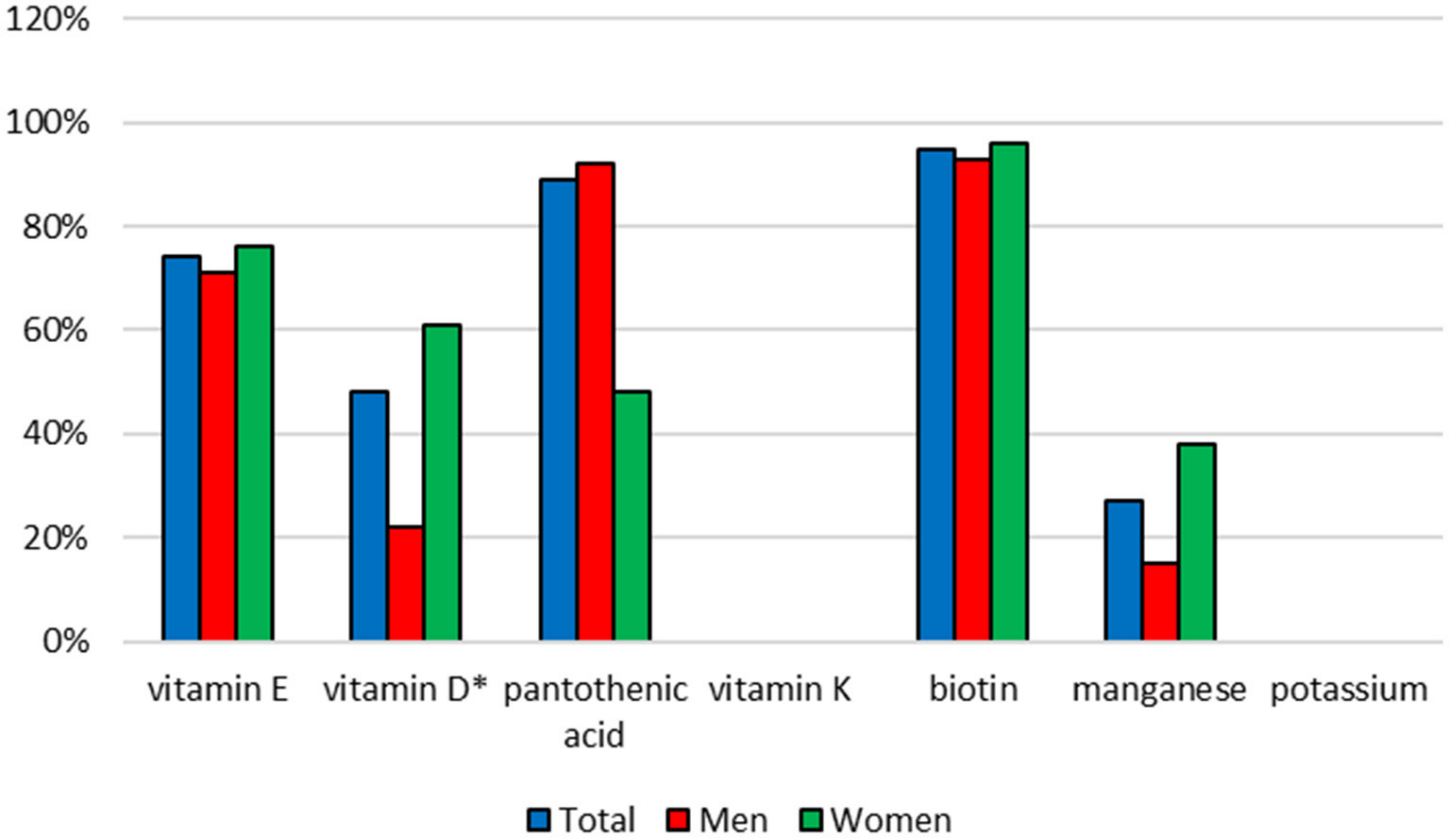
Percentage of people whose intake of vitamins or minerals from food supplements was higher than the AI value. *statistically significant difference, chi-square test, *p* < 0.05.

The vitamins and minerals intake was very differential. Intake of riboflavin, pantothenic acid and manganese was higher in the group of men (*p* = 0.0329; *p* = 0.0357; *p* = 0.0080, respectively) but intake of vitamin D was higher in the group of women (*p* = 0.0085) (Tables 3, 4). Food supplements were a significant source of vitamins and minerals in the diets of the respondents. In many cases, they covered the dietary reference values (EAR or AI) for these nutrients in 100%. Compared to men, intake of vitamin D higher than AI value in women was more likely (*p* = 0.0074) (Figures 4, 5). In each respondent it was calculated what percentage of the EAR or AI values was the intake of particular vitamins and minerals from food supplements. The percentage of EAR or AI was very differential and on average was ranged from 5% (potassium) to 478% (biotin). In some persons the vitamin intake was higher than the EAR or AI values several dozen times: biotin 83 times, vitamin B_12_ 50 times, vitamin C 31 times, riboflavin, niacin and vitamin B_6_ 23 times. A significant relationship was observed between gender and percentage of the EAR or AI values which was the intake of vitamin D (rs = 0.3661, *p* = 0.0055), calcium (rs = 0.3804, *p* = 0.0458), and copper (rs = −0.3994, *p* = 0.0288). In women, intake of vitamin D and calcium constituted a greater percentage of EAR or AI values, and intake of copper was a smaller percentage of EAR values than in men (*p* = 0.0108, *p* = 0.0479, *p* = 0.0389, respectively) (Table 5).

**Table 3:** Intake of vitamins from food supplements.

**Vitamins**	**Total**			**Men**			**Women**			**Men vs. women**
	**Median**	**Mean ± SD**	**Min–max**	**Median**	**Mean ± sd**	**Min–max**	**Median**	**Mean ± sd**	**Min-max**	*p*-value^*^
Vitamin A (RE. μg)	800.0	655.0 ± 182	300.0–800.0	600	612.5 ± 188.5	300–800	800	683.3 ± 180.1	400–800	0.3333
Beta carotene (μg)	200.0	1116.0 ± 1655.4	12.0–4000.0	200	1520.0 ± 1841.7	200–4,000	106	106.0 ± 132.9	12–200	1.0000
Vitamin E (TE. mg)	12.0	14.1 ± 14.0	3.6–100.0	12	18.1 ± 22.0	5–100	12	11.7 ± 4.9	3.6–30	0.5015
Thiamin (mg)	1.1	1.9 ± 3.8	0.3–25.0	1.4	3.1 ± 6.1	0.65–25	1.1	1.1 ± 0.3	0.33–1.7	0.0564
Riboflavin (mg)	1.4	2.1 ± 3.7	0.4–25.0	1.8	3.3 ± 6.0	0.75–25	1.4	1.4 ± 0.3	0.42–2.1	0.0329
Niacin (NE. mg)	16.0	17.7 ± 6.3	4.8–36.0	20	20.0 ± 7.4	8.5–36	16	16.2 ± 5.1	4.8–32	0.0651
Vitamin B_6_ (mg)	1.4	2.7 ± 4.5	0.4–30.0	1.4	3.4 ± 5.5	0.7–25	1.7	2.4 ± 4.1	0.4–30	0.9362
Folic acid (μg)	200.0	296.1 ± 198.3	60.0–800.0	200	278.0 ± 123.0	150–600	200	304.9 ± 227.4	60–800	0.5720
Vitamin B_12_ (μg)	2.5	5.0 ± 14.6	0.7–100.0	2.5	8.9 ± 24.3	0.67–100	2.5	2.8 ± 1.8	0.8–10	0.3368
Vitamin C (mg)	83.5	310.8 ± 455.5	15.0–2000.0	120	470.4 ± 586.9	15–2000	80	186.7 ± 267.4	24–1,000	0.0835
Vitamin D (μg)	10.0	27.5 ± 25.8	2.5–100.0	5	15.7 ± 20.4	2.5–60	50	33.1 ± 26.4	5–100	0.0085
Pantothenic acid (mg)	6.0	7.3 ± 3.9	1.8–25.0	7.5	9.4 ± 5.6	3–25	6	6.1 ± 2.0	1.8–12	0.0357
Vitamin K (μg)	30.0	33.2 ± 9.3	25.0–50.0	30	34.4 ± 9.8	25–50	30	31.7 ± 9.3	25–50	0.4777
Biotin (μg)	50.0	143.3 ± 388.9	15.0–2500.0	62.5	65.5 ± 22.8	25–112.5	50	185.2 ± 480.1	15–2,500	0.9639

*SD, standard deviation*;

* *Mann-Whitney U test*.

**Table 4:** Intake of minerals from food supplements.

**Minerals**	**Total**			**Men**			**Women**			**Men vs. women**
	**Median**	**Mean ± sd**	**Min–max**	**Median**	**Mean ± sd**	**Min–max**	**Median**	**Mean ± sd**	**Min–max**	*p*-value*
Calcium (mg)	162.0	256.5 ± 263.2	66.0–1000.0	162	196.7 ± 146.6	66–600	160	295.2 ± 315.3	80–1,000	0.9812
Magnesium (mg)	100.0	110.5 ± 68.6	30.0–400.0	100	119.4 ± 100.2	30–400	100	106.1 ± 47.1	56.3–300	0.5537
Iron (mg)	5.0	10.3 ± 13.3	1.0–56.0	5	7.1 ± 4.2	2.1–14	5.5	12.2 ± 16.3	1–56	0.8261
Zinc (mg)	12.5	7.1 ± 4.1	1.0–20.0	5	7.0 ± 5.3	1–20	6.3	7.2 ± 3.3	1.5–12.5	0.5058
Copper (mg)	0.5	0.6 ± 0.4	0.2–1.5	0.7	0.8 ± 0.3	0.5–1.5	0.5	0.5 ± 0.4	0.15–1	0.0536
Manganese (mg)	2.0	1.7 ± 0.9	0.6–4.0	2	2.2 ± 0.9	1–4	1	1.3 ± 0.6	0.6–2	0.0080
Iodine (μg)	100.0	118.8 ± 39.5	50.0–200.0	100	100.0 ± 20.4	75–150	125	129.2 ± 43.9	50–200	0.0613
Selenium (μg)	30.0	38.5 ± 21.5	8.0–100.0	34.1	45.4 ± 26.8	8–100	30	33.3 ± 15.5	8.3–55	0.2320
Phosphorus (mg)	125.0	136.2 ± 42.9	105.0–257.0	125	121.7 ± 8.2	105–125	125	158.0 ± 66.0	125–257	1.0000
Chrome (μg)	40.0	45.2 ± 38.4	6.0–200.0	40	47.1 ± 52.3	6–200	40	42.8 ± 8.3	25–50	0.1130
Molybdenum (μg)	50.0	47.1 ± 20.6	12.0–100.0	50	53.1 ± 20.9	25–100	50	37.4 ± 17.9	12–50	0.2124
Potassium (mg)	150.0	169.6 ± 127.8	40.0–320.0	150	200.0 ± 86.6	150–300	40	159.4 ± 141.8	40–320	1.0000

*SD, standard deviation*;

* *Mann-Whitney U test*.

**Table 5:** Percentage of EAR or AI which was the intake of vitamins and minerals from food supplements.

**Nutrients**	**Total**			**Men**			**Women**			**Men vs. Women**
	**Median**	**Mean ± SD**	**Min –max**	**Median**	**Mean ±SD**	**Min–max**	**Median**	**Mean ± SD**	**Min–max**	***p-*value**
Vitamin A	127	121 ± 38	48–160	111	112 ± 37	63–160	143	127 ± 39	48–160	0.3837
Vitamin E	150	159 ± 141	45–1,000	150	144 ± 66	60–375	150	170 ± 176	45–1,000	0.9824
Thiamin	122	184 ± 339	37–2,273	122	120 ± 16	100–150	122	214 ± 408	37–2,273	0.2053
Riboflavin	156	209 ± 333	47–2,273	156	153 ± 20	127–191	156	237 ± 410	47–2,273	0.3473
Niacin	145	155 ± 53	44–300	145	147 ± 30	107–200	145	158 ± 61	44–300	0.4253
Vitamin B_6_	127	219 ± 343	32–2,308	127	186 ± 184	50–909	143	235 ± 399	32–2,308	0.8621
Folic acid	59	74 ± 47	13–188	63	70 ± 36	44–178	44	76 ± 51	13–188	0.5959
Vitamin B_12_	125	249 ± 729	34–5,000	125	149 ± 109	40–500	125	294 ± 876	34–5,000	0.9710
Vitamin C	133	456 ± 655	20–3,077	133	559 ± 793	20–3,077	133	398 ± 566	20–2,667	0.8786
Vitamin D	67	183 ± 172	17–667	33	111 ± 131	17–333	333	227 ± 180	33–667	0.0108
Pantothenic acid	120	145 ± 78	36–500	128	138 ± 24	120–180	120	147 ± 90	36–500	0.5241
Vitamin K	46	55 ± 15	38–91	46	55 ± 20	45–91	55	55 ± 14	38–77	0.6993
Biotin	167	478 ± 1, 296	50–8,333	167	208 ± 64	167–333	208	607 ± 1, 570	50–8,333	0.4406
Calcium	16	27 ± 27	7–100	12	12 ± 3	8–16	16	32 ± 30	7–100	0.0479
Magnesium	38	38 ± 22	9–114	34	36 ± 25	9–113	38	39 ± 21	9–114	0.3086
Iron	83	155 ± 191	13–933	124	122 ± 105	13–375	83	172 ± 224	13–933	0.7449
Zinc	74	94 ± 53	11–213	90	93 ± 62	11–184	74	95 ± 48	22–213	0.7613
Copper	71	86 ± 53	21–214	143	108 ± 49	21–143	71	69 ± 51	21–214	0.0389
Manganese	87	84 ± 36	33–174	87	86 ± 19	56–111	71	83 ± 44	33–174	0.4838
Iodine	105	125 ± 42	53–211	105	129 ± 40	79–211	105	123 ± 43	53–211	0.6888
Selenium	67	86 ± 48	18–222	78	88 ± 34	18–122	67	84 ± 55	18–222	0.2909
Phosphorus	22	23 ± 7	18–44	22	21 ± 2	18–22	22	24 ± 9	18–44	1.0000
Potassium	4	5 ± 4	1–9	4	4 ± 3	1–9	9	6 ± 4	1–9	0.5303

SD, standard deviation; *Mann-Whitney U test.

The intake above UL in a few cases relating to vitamin B_6_-1 person (woman) and magnesium −3 persons (two men and one woman) was observed. Moreover intake of vitamin B_6_ and vitamin D was equal to the UL values, respectively in one man and two women.

## Discussion

According to our data from the survey presented in this manuscript the rate of adults using food supplements in the Polish population in 2019–2020 was 6% in men and 13% in women. These data apply only to preparations classified as food supplements. Preparations classified as OTC drugs were not included in this analysis.

In 2000, the first representative survey of the dietary intake of the entire population were carried out in Poland (21). These studies also estimated the frequency of using food supplements without taking OTC drugs into account. The survey from 2000 showed that food supplements were used by 11% of men, and 18% of women (29, 30).

Both in our survey from 2019–2020 and in the cited studies from 2000 (21), food supplements were used more often by females than by males.

In the survey from 2000, it was shown that supplementation did not significantly increase the average intake of most minerals and vitamins in the population. It increased only the average intake of iron, nicotinic acid, vitamin B_6_ and vitamin E in women and vitamin E in men (29).

According to our survey from 2019–2020 the percentage of respondents using food supplements was small. However, in the diet of users, supplements were an important source of nutrients.

Vitamins B_6_, C, D and magnesium were nutrients most often taken in the form of food supplements. Vitamin B_6_, D and magnesium were taken more frequently by women than men. The average content of most nutrients in food supplements was quite high compared to the Dietary Reference Values (DRVs). However, the amounts of calcium, magnesium, phosphorus and potassium taken from supplements were not so high compared to the DRVs. Taking into account the median, the amount of vitamin D in men's diets from supplements was much lower than AI.

Certain population groups in Poland may use food supplements much more often (31–35). For example, in the years 2011–2012, dietary intake studies were carried out, taking into account food supplement use in Warsaw population aged 20–74 years. Subjects supplemented their diets with vitamins and minerals quite often. The rate of food supplement users was higher in women (40%) than in men (31%). Subjects with higher education level and the highest income used supplements more often than those with primary education or the lowest income. Using food supplements containing vitamin D, thiamine and folic acid contributed to better coverage of the requirements for these vitamins (31).

Another survey conducted in 2012 in students of the University of Rzeszow showed that the majority of respondents − 71.5% used food supplements. However, supplements have not always been used regularly; almost 40% of men and 30% of women consumed it occasionally. The most commonly used were food supplements containing vitamins, less often those containing minerals (32).

The use of supplements was also frequent in the group of subjects consisting mainly of young people, including the students of Medical University of Lublin. Food supplement consumption was reported by 77.84% of participants, however, 24.82% of women and 28.57% of men used them regularly. The subjects consumed mainly products containing only vitamins or vitamins and minerals, less probiotics and products containing only minerals (33).

In 2015 study conducted among 205 Polish adults (general population) and 49 pregnant women or planning pregnancy showed that 54% of respondents of both sexes from the general population used food supplements. About half of the respondents declared consumption of food supplements every day. Food supplements were more often used by pregnant women and those planning to become pregnant (89 and 56%, respectively) (34). Frequent food supplement consumption was also observed in another study in the group of women during pregnancy and women becoming pregnant (82 and 41%, respectively) (35). In our study pregnant women were not included.

Data on food supplement use in Poland vary depending on the group. Surveys of the representative population for the country: from 2000 (29) and described in this manuscript from 2019–2020 showed a lower proportion of supplement users than surveys of target groups (31–35). However, the differences in data may be due to the fact that some authors could include OTC drugs containing vitamins and minerals into supplements.

Surveys conducted in Poland show that women use food supplements more often than men (29, 31), which was also confirmed by our study. Also, higher income, higher education level and living in large cities are conducive to using food supplements (31, 34). The relationship between the level of education and consumption of supplements may be confirmed by the relatively high proportion of food supplement users among students (32, 33).

In our study, the majority of supplement users (80% of males and 71% of females) consumed them every day. Less regularity was noted in other groups (32, 33). Both in the nationwide population and in other studied groups, products containing vitamins and/or minerals were used most often (29, 32, 33). Food supplements most often provided vitamin D and magnesium (31, 34).

Studies conducted in various European counties showed that the level of food supplement consumption was differential (from 8 to 60%). Food supplements were most often taken by the Danes (51% of men and 60% of women). Supplementation was also frequent in adult Finns (32 and 58%, respectively), Italians (41 and 56%, respectively) and Germans (38 and 48%, respectively). Spain had the lowest percentage of adults using supplements (8% men and 10% women). Results of our survey showed that frequency of food supplement consumption in Poland was low comparing to other European countries (30, 36–38).

In the United States, in 2007–2010, 49% of adults reported using food supplements, it was more common in women than men. Older adults reported higher use than younger. Multivitamin-mineral products were the most common type of food supplements reported (16).

A study conducted in Novi Sad in Serbia in 2019 showed that 42.8% of respondents were using food supplements. Females were more likely to use supplements than males. Minerals, vitamins or their combinations were the most commonly used supplements (68.8% of users). Contrary to the results of other studies, respondents with minimal and average incomes were more likely to use supplements compared to those with incomes above average (38).

In 2012–2013 there was investigated which nutrients are most often taken with supplements in Germany. The most frequently were consumed supplements containing vitamins: C (53% of all supplement users), E (45%) and B (37–45%) and minerals: magnesium (59.2%), calcium (37.0%), zinc (33.6%), and selenium (23%) (39, 40). In our survey, these nutrients were also often taken with supplements. However, supplements containing vitamin D and iron were also frequently used, while those containing calcium were consumed slightly less often.

As in Poland, in some countries survey on the consumption of food supplements was carried out in selected population groups.

In the United Arab Emirates (UAE) in 2018 the prevalence of food supplement use in college students was 35.6%. Supplements were used more often by males than females. Protein, and vitamins D and C were the most commonly used by males. Females consumed mostly multi-vitamins and/or minerals, and single vitamins (41).

In Poland and other European countries, women use supplements more often (30, 36, 37). Higher income does not always favor the use of supplements, as shown by studies from Serbia (38). In all countries, products containing vitamins and/or minerals are the most commonly used (30, 36–38).

It is very important to determine what nutrients are provided with the supplements and in what amounts. For this reason, it was a key element of our survey. However it is difficult to compare our data on vitamin and mineral intake from food supplements with the data of other authors. We analyzed what amounts of individual nutrients came from food supplements. The published data usually indicated mean intake of nutrients from food and from food and food supplements together (including food supplement users and non-users together) (30) or mean intake of nutrients from supplements only but in the group of all users (regardless used supplements contained an individual nutrient or not) (39, 40).

Supplementing the diet with vitamins and minerals in many cases may contribute to a better implementation of nutritional recommendations. However, consumers should be aware of the risk related to excessive intake of vitamins and minerals. Food supplements contain active ingredients that provide a physiological effect but also may cause adverse effect, especially in susceptible people (22). Some individuals use multiple supplements, which can result in potential interactions between them.

In our survey in some respondents, the intake of vitamins such as biotin, vitamin B_12_, C, riboflavin, niacin and vitamin B_6_ was several dozen times higher than the DRVs. In addition, in several subjects the intake of vitamin B_6_, D or magnesium was equal to or higher than the UL values. Moreover, some food supplement users could be at risk of interactions between the ingredients of supplements. 24% of respondents declared using more than one supplement during the day.

In the aforementioned survey in Germany a few subjects reached or exceeded the UL values through supplements alone. It concerned vitamin A, calcium, magnesium and zinc (39, 40).

Our research was conducted just before the COVID-19 pandemic and it is worth to see how the pandemic might affect supplement intake. As indicated the PLifeCOVID-19 Online Studies the intake of food supplements was influenced by the COVID-19 pandemic. The supplementation was initiated more frequently during the first than the second wave of the pandemic. Supplement users were primarily females, younger and better educated persons, living with the family, residents of urban areas. The most frequently supplemented nutrients were vitamin D, vitamin C, omega-3 fatty acids, folic acid, and magnesium. Compared to the pre-pandemic period, the consumption of supplements such as vitamins C and D, zinc, garlic, ginger, or turmeric has increased. Improving immunity was the main reason to use food supplements (42).

However, current guidelines for the treatment of COVID-19 do not suggest using food supplements (43). The decision to use food supplements should be preceded by a consultation with a physician or dietitian. Supplements are often used by people who care for a proper diet, which can provide them with adequate amounts of vitamins and minerals. In such cases, supplementation is not always necessary (44).

It is important to assess whether the diet is deficient in nutrients, in which nutrients and how the deficiency can be eliminated e.g., by modifying the diet, eating fortified food or using supplements. An assessment of the importance of food supplements in covering the requirements for individual nutrients in relation to its intake from all sources (food, fortified food, and supplements) is also crucial. The potential risk of adverse effects and interactions should also be considered.

There are several limitations regarding our survey. Analyses of mineral and vitamin intake from supplements concern subjects who used food supplements. As the percentage of them in the study population was small, the sample size was 178 subjects. However, the percentage of supplements users (10%) was estimated on the basis of data for 1,831 respondents. In addition, the survey did not take into account all the factors associated with using supplements, including respondents' knowledge about supplements and the reasons for supplementation.

The survey has many strengths. The rate of supplement users was assessed in a group representative for the adult population in Poland. The data was collected using two methods (food propensity questionnaire and repeated 24-h recall), which strengthens their credibility. The analyses concern only food supplements, but do not take into account OTC drugs. Detailed data on the name of the preparation and dose used was collected from each of the subjects. The survey provides relevant findings on vitamin and mineral intake with a warning around risk for excessive intakes.

## Conclusions

According to the data collected a minority of adults in Poland declared the use of food supplements during the year. However, most of the supplement users consumed such products every day and food supplements were a significant source of vitamins and minerals. In this survey the gender of the respondents had an impact on the type of supplement used, the type of supplemented vitamins and minerals, as well as the intake and coverage of Dietary Reference Values in case of some of these nutrients. In some persons the intake of biotin, vitamin B_12_, C, B_6_, riboflavin, niacin was higher than the reference values several dozen times. The intake above UL in a few cases relating to vitamin B_6_ and magnesium was observed. Due to the risk of excessive intake of vitamins and minerals from food supplements in combination with the diet, the consumption of these products should be monitored.

## Data Availability Statement

The raw data supporting the conclusions of this article will be made available by the authors, without undue reservation.

## Ethics Statement

The studies involving human participants were reviewed and approved by the Bioethics Committee at the Institute of Food and Nutrition in Warsaw (opinion dated 04.06.2018). The patients/participants provided their written informed consent to participate in this study.

## Author Contributions

KS conceived the study, defined basic theses, wrote, and revised final version of the manuscript. AW contributed to study design, performed the statistical analysis, interpretation of the results, and wrote the manuscript. ER did the literature review and wrote the manuscript. IZ prepared data for analysis and wrote the manuscript. AG and MO prepared data for analysis. All authors contributed to the article and approved the submitted version.

## Funding

The data for this paper came from the survey financially supported by European Food Safety Authority (No. OC/EFSA/DATA/2015/03 CT 2 and No. OC/EFSA/DATA/2015/03 CT 3) and Polish Ministry of Science and Higher Education (No. 3876/E-220/S/2018-1). This study was also performed under the project of National Institute of Public Health NIH – National Research Institute, Poland (FŻ-1/2021).

## Conflict of Interest

The authors declare that the research was conducted in the absence of any commercial or financial relationships that could be construed as a potential conflict of interest.

## Publisher's Note

All claims expressed in this article are solely those of the authors and do not necessarily represent those of their affiliated organizations, or those of the publisher, the editors and the reviewers. Any product that may be evaluated in this article, or claim that may be made by its manufacturer, is not guaranteed or endorsed by the publisher.
